# Septic Arthritis and Bacteremia Due to Infection by Pasteurella canis

**DOI:** 10.7759/cureus.19478

**Published:** 2021-11-11

**Authors:** Bruna Nascimento, Ana Garrido Gomes, Carolina Nunes Coelho, Marta Guisado, Ramona-Diana Bindean

**Affiliations:** 1 Internal Medicine, Hospital Distrital de Santarém, Santarém, PRT; 2 Clinical Pathology, Hospital Distrital de Santarém, Santarém, PRT

**Keywords:** pasteurella infections, zoonoses, bacteremia, septic arthritis, pasteurella canis

## Abstract

*Pasteurella canis *is a Gram-negative coccobacilli from the *Pasteurellaceae* family. The most common form of transmission to humans is a bite from a dog or a cat. We report a case of a 90-year-old woman who presented with septic arthritis in the right knee and bacteremia two weeks after a cat bite. The patient was treated with arthrocentesis and directed antimicrobial therapy. Human *Pasteurella canis *infection is a rare occurrence, making this a case of note.

## Introduction

*Pasteurella* spp. are nonmotile, facultatively anaerobic, Gram-negative coccobacilli which belong to the ​*Pasteurellaceae *family [1]. *Pasteurella* spp. have a worldwide distribution and are common commensals of the oral cavity and gastrointestinal tract of many animals. In humans, the infection is most often caused by dog and cat bites but it can also develop in patients exposed to animals without a history of bites or scratches [1].

Human infection by *Pasteurella* spp. may result in cellulitis, subcutaneous abscesses, arthritis, osteomyelitis, pneumonia, pleural effusion, meningitis, bacteremia, endocarditis, and peritonitis [1,2]. *Pasteurella multocida* is the most common pathogen found in human infection, but we can also find reports of infection caused by *Pasteurella canis*, *Pasteurella dagmatis, *and *Pasteurella stomatis *[2].

Human infection by *Pasteurella​​​​​​​ canis* is rare and there are reported cases of soft tissue infections [3-7], bacteremia [5,7-9], eye infections [10,11], respiratory infection [8,9,12], septic arthritis [13,14], osteomyelitis [3,15], gastrointestinal infection [16], breast implant infection [17], and peritonitis [18]. The first-line treatment is with penicillins and alternatively with fluoroquinolone, doxycycline, or trimethoprim-sulfamethoxazole [1].

## Case presentation

**Figure 1:**
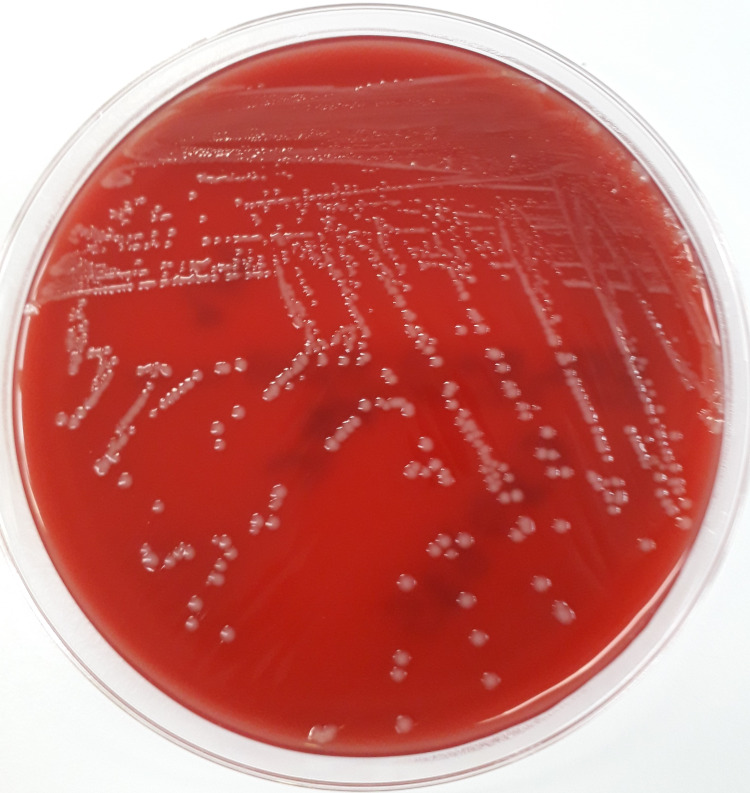
Colonies of Pasteurella canis isolated from the articular fluid.

A 90-year-old Caucasian woman was admitted to the internal medicine ward with a history of pain in the right knee and hypotension. She had previous diagnoses of diabetes mellitus with nephropathy, severe aortic stenosis, arterial hypertension, atrial fibrillation, and arthrosis of both knees. The patient lived alone in the countryside and had a contact history with dogs and stray cats that she used to feed. Upon physical examination, the blood pressure was 90/45 mmHg, the temperature was 38.5ºC, and the right knee presented with swelling, pain, and loss of function with walking limitation. Laboratory examination revealed leukocytosis with predominance of neutrophils and a C-reactive protein of 14 mg/dL (normal<0.5 mg/dL). Two sets of blood cultures were collected and an arthrocentesis was performed with drainage of cloudy fluid. Empirical treatment with piperacillin and tazobactam adjusted to the renal function was initiated. The cytological examination of the articular fluid revealed 127,000/uL white blood cells with 90% of polymorphonuclear cells, compatible with septic arthritis. The bacteriological examination of the articular fluid and blood cultures revealed grayish-white, mucoid, nonhemolytic colonies compatible with *Pasteurella *spp. and the automatic bacterial identification system confirmed the presence of *Pasteurella canis *(Figure 1). After this result, we retraced the anamnesis and the patient recalled a cat bite two weeks before. A transthoracic echocardiogram was performed and did not show signs of endocarditis. Because of the favorable clinical course, past medical history, and negative blood cultures after 14 days of treatment, we opted not to perform a transesophageal echocardiogram. No other infection foci were detected. The antibiotic scheme was de-escalated to amoxicillin with clavulanic acid and the patient completed a total of three weeks antibiotic course with good clinical response.

## Discussion

Human infection with *Pasteurella* spp. can be divided into three groups - infection after animal bites (mostly from dogs or cats), infection after other animal exposures, and infection with no animal contact [1]. *Pasteurella canis *is the most common species isolated from dog bites [19] but it can also be found in cases of cat bites [12,20]. Infection after a dog or cat bite is the most common, and in our report, the transmission may have been either from the cat bite or from close contact with the dog.

*Pasteurella *spp. severe infections can occur in healthy individuals but children, the elderly, and those with immunocompromised conditions are at greater risk. For septic arthritis, prosthetic joint increases the risk of infection [2]. Specifically, in infection due to *Pasteurella canis, *there are reports in diabetic [3,4] and immunocompromised patients [5,10,16]. Our patient had risk factors like advanced age and a history of diabetes but she had no knee prosthetics.

There are multiple case reports of bacteremia due to infection by *Pasteurella canis *[5,7-9] but there are few reports of septic arthritis, being this case the first one reporting knee involvement [13,14]. Penicillins like penicillin G, penicillin V, ampicillin, amoxicillin, amoxicillin with clavulanic acid, and piperacillin with tazobactam have a good *in vitro* activity against *Pasteurella *spp. Later generation of cephalosporins like cefuroxime, cefixime, ceftriaxone, and ceftaroline also demonstrate great *in vitro *activity. Alternatively, for patients intolerant to beta-lactam, fluoroquinolone, doxycycline, or trimethoprim-sulfamethoxazole may be a good option [1]. Duration of antimicrobial treatment is the same as for other kinds of infections [2]. Initially, the patient was empirically treated with piperacillin with tazobactam because she presented signs of severe infection. With the isolation of *Pasteurella canis *in the hemocultures and articular fluid, we changed the antibiotic to amoxicillin plus clavulanic acid administered intravenously during 21 days. Treatment of septic arthritis should consist of frequent drainage along with antimicrobial therapy [1,2]. Drainage of the articular fluid was performed and consequently we observed a favorable evolution with reduction of the inflammatory signs and recovery of some functionality of the knee.

For patients with bacteremia due to *Pasteurella *spp. the mortality rate is high, but for those with septic arthritis, the prognosis is good with full recovery [1]. Specifically, bacteremia due to *Pasteurella canis *has a favorable prognosis and our patient survived in spite of advanced age and associated comorbidities [5,7-9]. In the reported cases of septic arthritis, the patients presented with complete resolution of symptoms but our patient had a slow functional recovery of the knee despite physical rehabilitation, and at the time of hospital discharge, she wasn't able to walk without support [13,14]. This unfavorable prognosis may be due to her history of arthrosis. However, there is no support in literature about this aspect in infection due to *Pasteurella canis*.

## Conclusions

Human infection by *Pasteurella canis *is not common but this agent and others from the *Pasteurella *genus should be considered in patients with a history of animal bite, and more severe infections may require hospital admission and surgical intervention. We reported the case of a 90-year-old woman with a personal history of diabetes who presented with septic arthritis and bacteremia. She was treated with arthrocentesis and antimicrobial therapy with good clinical response. This case attests to the need for a comprehensive clinical history focusing on animal exposure, so as to be more attentive to infections by these kinds of pathogens.
